# Contractile Response of Bovine Lateral Saphenous Vein to Ergotamine Tartrate Exposed to Different Concentrations of Molecularly Imprinted Polymer

**DOI:** 10.3390/toxins10020058

**Published:** 2018-01-30

**Authors:** Manoj B. Kudupoje, James L. Klotz, Alexandros Yiannikouris, Karl A. Dawson, Kyle R. McLeod, Eric S. Vanzant

**Affiliations:** 1 Department of Animal and Food Sciences, University of Kentucky, Lexington, KY 40546, USA; mbku223@uky.edu or mkudupoje@alltech.com (M.B.K.); kyle.mcleod@uky.edu (K.R.M.); 2 Center for Animal Nutrigenomics & Applied Animal Nutrition, Alltech Inc. 3031 Catnip Hill Road, Nicholasville, KY 40356, USA; ayiannikouris@alltech.com (A.Y.); kdawson@alltech.com (K.A.D.); 3 USDA-ARS, Forage-Animal Production Research Unit, Lexington, KY 40546, USA; james.klotz@ars.usda.gov

**Keywords:** ergot alkaloids, myograph, imprinted polymer

## Abstract

Ergot alkaloids, in their active isomeric form, affect animal health and performance, and adsorbents are used to mitigate toxicities by reducing bioavailability. Adsorbents with high specificity (molecularly imprinted polymers: MIP) adsorb ergot alkaloids in vitro, but require evaluation for biological implications. Using ex vivo myography, synthetic polymers were evaluated for effects on the bioactivity of ergotamine tartrate (ETA). Polymers were first evaluated using isotherms. Lateral saphenous veins were collected from 17 steers for four independent studies: dose response of ETA, adsorbent dose response, validation of pre-myograph incubation conditions and MIP/ non-molecularly imprinted polymer (NIP) comparison. Norepinephrine normalized percent contractile response to increasing ETA exhibited a sigmoidal dose response (max: 88.47 and log of the effective molar concentration (EC_50_) (−log [ETA]) of 6.66 ± 0.17 M). Although sample preparation time affected contractile response (*p* < 0.001), pre-myograph incubation temperature (39 vs. 21 °C, 1 h) had no effect (*p* > 0.05). Isothermal adsorption showed a maximum adsorption of 3.27E-008 moles·mg^−1^ and affinity between 0.51 and 0.57 mg (*R*^2^: 0.83–0.92) for both polymers, with no significant difference between polymers (*p* > 0.05). No significant differences in maximum inhibitory (*p* = 0.96) and IC_50_ responses (*p* = 0.163) between MIP and NIP were noticed. Normalized percent contraction could be predicted from the in vitro adsorption data (*R*^2^ = 0.87, *p* < 0.01), for both polymers. These studies indicate that synthetic polymers are potentially effective adsorbents to mitigate ergot toxicity caused by ergot alkaloids, with little evidence of significant differences between MIP and NIP in aqueous media.

## 1. Introduction

Alkaloids produced by the endophytic fungus *Epichloë coenophiala* are known to produce complex biological responses in animals due to agonistic and antagonistic interactions with biogenic amine receptors [1]. Ergot alkaloids can induce strong and prolonged vasoconstriction in human coronary and temporal arteries [2], in canine saphenous and femoral veins [3], and in arterial preparations from rats and guinea pigs [4]. Myographic studies using several ergot derivatives have confirmed the vasoconstrictive effects in bovine preparations of lateral saphenous veins [5], right ruminal arteries and veins [6], and mesenteric arteries and veins [7]. Studies have also established interactions between ergot alkaloids and neurotransmitter receptors, indicating the structural similarities between the alkaloids and biogenic amines (neuro-hormones), like serotonin [8,9], norepinephrine [10,11] and dopamine [12,13], as a cause for vasoconstriction. Additionally, among two structural classes of ergot alkaloids, ergopeptides (e.g., ergotamine, ergocornine, ergocryptine and ergocristine) are known to produce more persistent dose dependent vasoconstriction, while ergolines (e.g., ergonovine and lysergic acid) produce rapid, but less intense and less persistent, contractile responses [14].

Ergot alkaloids contain a C9=C10 double bond that can readily epimerize with respect to the stereogenic center at C-8, especially in the presence of alkalis, forming a series of right-hand rotation (S)-isomers representing isolysergic acid derivatives [15]. The C-8 epimers differ in biological and physicochemical properties and have different pKa values [16,17]. Studies have shown that C-8-(R) isomers (-ines) are biologically active, while the C-8-(S) isomers (-inines) are inactive [1,18]. The conversion of -ines to -inines is rapid, especially in aqueous acidic or alkaline solutions [15] and the -inines are likely to be reactivated to their biologically active -ine form [19]. Studies have shown that ergopeptinines can also convert back into the -ine form, especially in methanol, aqueous organic solvents and acids [20]. Additionally, ergot alkaloids are sensitive to light [15,21] and heat [22,23], which leads to both isomerization and degradation. Because epimerization influences biological activity, when evaluating remediation approaches, the use of bioassays may provide information that would not be available from simple quantification of alkaloid concentrations.

Researchers have evaluated the application of toxin adsorbents, including esterified glucomannans, commercially available clay-based products [24] and yeast cell wall based products (FEB-200^TM^: [25]), to alleviate the negative effects of endophyte toxins, and have indicated a reduction in the bioavailability of toxins in the gastrointestinal tract (GIT). In recent years, adsorbents synthesized using molecular imprinting technologies have gained importance due to specific binding through selective recognition. Molecular imprinting is a technique for synthesizing macromolecular polymers with multi-functional receptor groups that aid in specific interaction with a targeted molecule [26,27,28]. Generally, molecularly imprinted polymers (MIP) are synthesized using functional monomers, cross-linkers, an initiator and porogen in the presence of an adsorbate of interest, called a template. The template interacts with functional monomers during polymerization, which are then networked with cross-linkers to form polymers of larger molecular weight (generally > 1000 kDa, depending on the monomer type) [29,30]. These polymers are then washed to remove the template, thereby creating heterogeneous binding sites with functional groups arranged in geometries capable of binding the template and similar molecules with varying affinities [31]. Because of specific molecular recognition properties, MIPs have been applied as sorbent materials for sample clean-up in analytical chemistry [32] and as sensors in biomedical applications [33]. However, to date, there have been no studies with the application of synthetic polymers as toxin adsorbents in animal feed.

Ergovaline is the predominant alkaloid in naturally contaminated tall fescue and is found in the range of 0.5 to 5 mg·kg^−1^ [34]. Studies have suggested that ergotamine concentrations in contaminated forage and seeds are generally found at a rate of 0–12 and 15–40 percent of ergopeptides, respectively [35,36]. Levels of total ergopeptides in surveys of rye have been reported at over 7 mg·kg^−1^ [37], with ergotamine as the major alkaloid. These studies suggest that ergotamine is comparatively greater in *Clavicep* infection (rye) while ergovaline is in greater concentrations in *Epichloë* infection (tall fescue).

To understand how best to use adsorbents to help mitigate ergot alkaloid toxicity, the effects must be studied in a biological system. The kinetics of adsorbent-adsorbate interactions are profoundly influenced by the equilibrium conditions. The in vitro efficacy of adsorbents may be erroneously interpreted with respect to their biological implications, due to the simplified nature of in vitro experimental models, without encountering biological events that could change the efficacy, and also without accountability for isomeric forms and external factors that influence biological responses. Therefore, as a complement to in vitro studies, ex vivo bioassays have been used as means to bridge the gap between in vitro studies and complex in vivo experiments. The interactions between ergot alkaloids and neurotransmitters has been extensively studied using myograph-based techniques [5,6,38,39]. Myographic bioassays are based on the contractile response of smooth muscle present in blood vessels. Ergotamine and ergovaline have been shown to produce dose dependent smooth muscle contraction of blood vessels [5].

In vitro isothermal adsorption studies are commonly used to evaluate adsorbents for their adsorption efficiency. However, the biological implications of in vitro adsorption studies need to be established and the understanding of consequences of alkaloid epimerization during the experimental procedures in the presence of polymers has to be assessed. To aid understanding of the biological implications of adsorbing dietary alkaloids with a synthetic, molecularly-imprinted polymer, our main objective was to determine the extent to which the ex vivo myographic response to ergotamine in the presence of polymers would correspond to predictions from isothermal adsorption studies. Ergotamine tartrate was used as a template molecule because it is a commercially available molecule with stereochemistry that is representative of the ergot peptide family, and also because of the existence of well-established analytical methods for quantification [18,37]. Previously synthesized and characterized MIP and corresponding non-molecularly imprinted polymer (NIP) [40,41] were used as adsorbents.

## 2. Results

Prior to evaluating the ability of polymers to mitigate the physiological effects of ergotamine tartrate (ETA), we conducted a series of experiments to establish (1) the myographic dose response to ETA, and (2) the proper incubation conditions for the evaluation of polymer effects.

### 2.1. Dose Response to ETA

**Figure 1:**
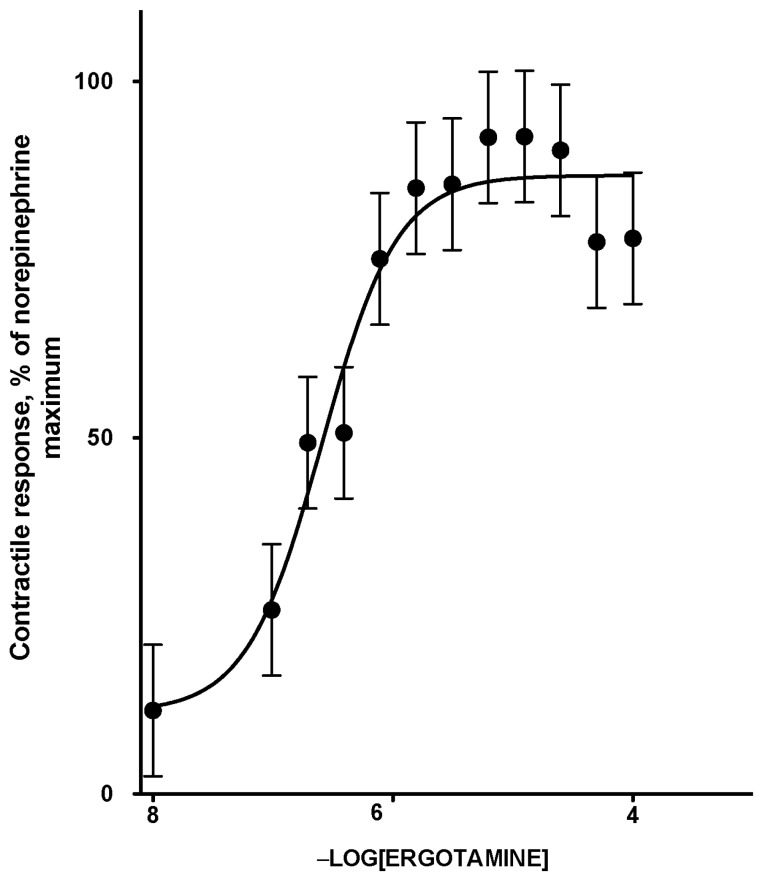
Norepinephrine normalized contractile response curve of smooth muscle (lateral saphenous vein) to increasing concentrations of ETA. The points are means and the vertical bars show the SEM (*n* = 10). Percent contraction (NE normalized) = 88.5 + ((5.7 − 88.5)/(1 + 10^6.664−intital [ETA]^)) (Equation (3), Section 5.7).

The contractile response of lateral saphenous vein cross sections to logarithmically increasing concentrations of ETA was described by a sigmoidal relationship (*R*^2^ = 0.95; Figure 1; Table 1). The relationship was linear between 9.766 × 10^−8^ and 6.250 × 10^−6^ M ETA, and the maximum contractile response of 88% was observed at and above 3.125E-06 M ETA. The effective molar concentration (EC_50_) of ETA that produced 50 percent of the norepinephrine (NE)-normalized contractile response in terms of −log [ETA] was 6.66 ± 0.17 (2.26 × 10^−7^ M). The results were comparable to −log EC_50_ values of 6.48 ± 0.38 M obtained for mesenteric veins from steers fed endophyte free tall fescue seeds, when exposed to ETA [7]. Although the range of ETA concentrations used to construct the dose response curve may have exceeded levels expected to be physiologically relevant, these concentrations were appropriate for achieving saturation of receptor-agonist interactions to allow accurate calculation of the EC_50_.

### 2.2. Validation Studies

**Figure 2:**
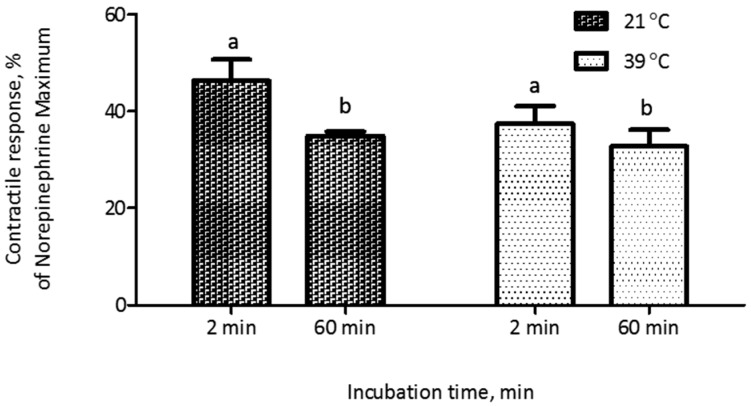
Effect of incubation temperature and length of incubation in buffer containing ETA (7.813 × 10^−7^ M) on the contractile response of saphenous veins. Each data point was the mean of six replications of blood vessels ± SEM. Incubation time (60 min) reduced (*p* < 0.001) the contractile response and there was no effect (*p* > 0.05) of temperature or interaction between incubation time and temperature (*p* = 0.26). Different letters (a,b) above columns denote significant differences (*p* < 0.001) between incubation times.

**Figure 3:**
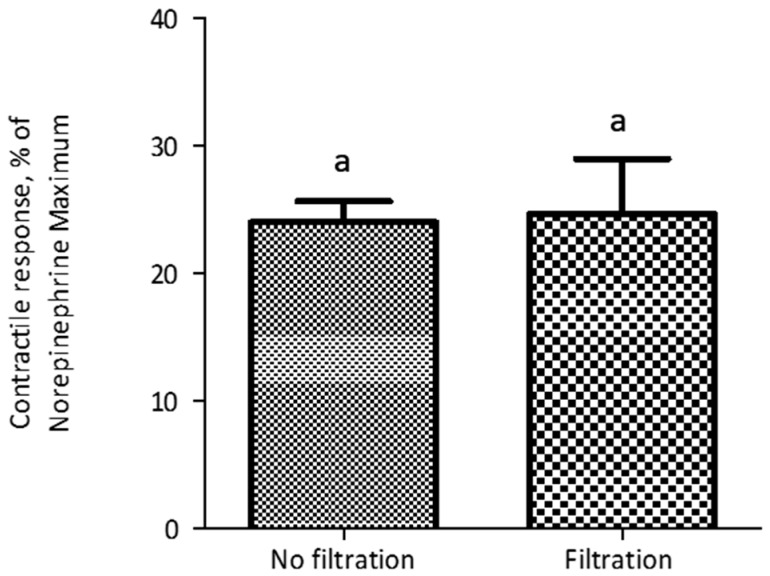
The contractile response of lateral saphenous veins exposed to ETA treatment (7.813 × 10^−7^ M) that were filtered through polypropylene filtration columns fitted with quartz frits, at 39 °C. Each data point represents mean of six replications of blood vessels ± SEM. There was no significant difference (*p* = 0.8752) in response between the filtration and no filtration methods. Similar letters (a) above columns denote lack of significant difference (*p* > 0.05) between columns.

The evaluation of the polymers introduced an additional preparatory step before myographic analysis, namely the incubation of polymers with ETA. Thus, we conducted experiments to evaluate the potential influence of conditions associated with this incubation on the contractile response of blood vessels. In a feed additive application, polymers would be exposed to alkaloids within the GI tract of an animal, at a temperature of around 39 °C. Thus, we compared incubation at typical room temperature (21 °C) with incubation at 39 °C. Additionally, we recognized that the incubation time could result in substantial differences in adsorption, and that these effects might interact with the incubation temperature. Thus, we constructed a 2 × 2 factorial treatment structure to evaluate each combination of these two incubation temperatures and two incubation times (2 min and 60 min). Results indicated that an incubation time period of 60 min prior to myographic evaluation reduced (*p* < 0.001) the contractile response, compared to a 2-min incubation period, when ETA solution was incubated either at 21 °C or 39 °C, with no interaction (*p* = 0.26) between incubation time and temperature. Additionally, there was no significant difference (*p* > 0.05) in contractile response due to incubation temperature (Figure 2). The standard adsorption evaluation protocol included 60 min of incubation and 30 min of centrifugation. To reduce the potential time effect associated with centrifugation, an alternate protocol was evaluated, in which the centrifugation period of 30 min (25,000 *g*, 39 °C) used to separate bound and free ETA was replaced with filtration (polypropylene filtration columns fitted with quartz frits). Before implementing the new protocol, non-specific binding of ETA on filtration columns was evaluated. There was no significant difference (*p* = 0.87) in myographic response between the ETA treatment (7.813 × 10^−7^ M) that was filtered through the filtration column and the control (without column filtration; Figure 3). It was observed that the myographic response decreased by 25 percent when the incubation period was 60 min at each temperature (21 °C vs. 39 °C) at similar ETA concentrations.

### 2.3. Polymer Evaluation: Isothermal Adsorption Studies

**Figure 4:**
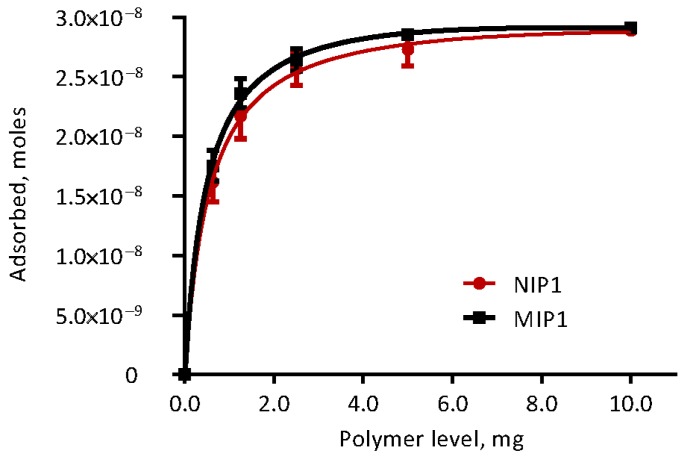
Ergotamine adsorption of polymers (molecularly imprinted polymers, MIP and non-molecularly imprinted polymer, NIP), in modified Krebs–Henseleit buffer media fitted to binding saturation curve with specific binding sites.

The isothermal adsorption properties of polymers to ETA in modified Krebs-Henseleit buffer were evaluated. The proportional amount of ETA adsorbed on the polymers with different inclusion rates of MIP and NIP are shown in Figure 4, and the adsorption parameters derived from a one-site total binding curve are included in Table 2. The goodness of fit (*R*^2^) was better for MIP (0.93) compared to NIP (0.84). Maximum ETA adsorption (B_max_) occurred when the polymer inclusion rate exceeded 5 mg in 10 mL of incubation media, and did not differ (*p* = 0.78) between polymer types. Similarly, neither dissociation constants (*K_d_*), nor slopes of nonspecific binding (NS), differed between MIP and NIP (*p* ≥ 0.80).

### 2.4. Polymer Evaluation: Myographic Studies

**Figure 5:**
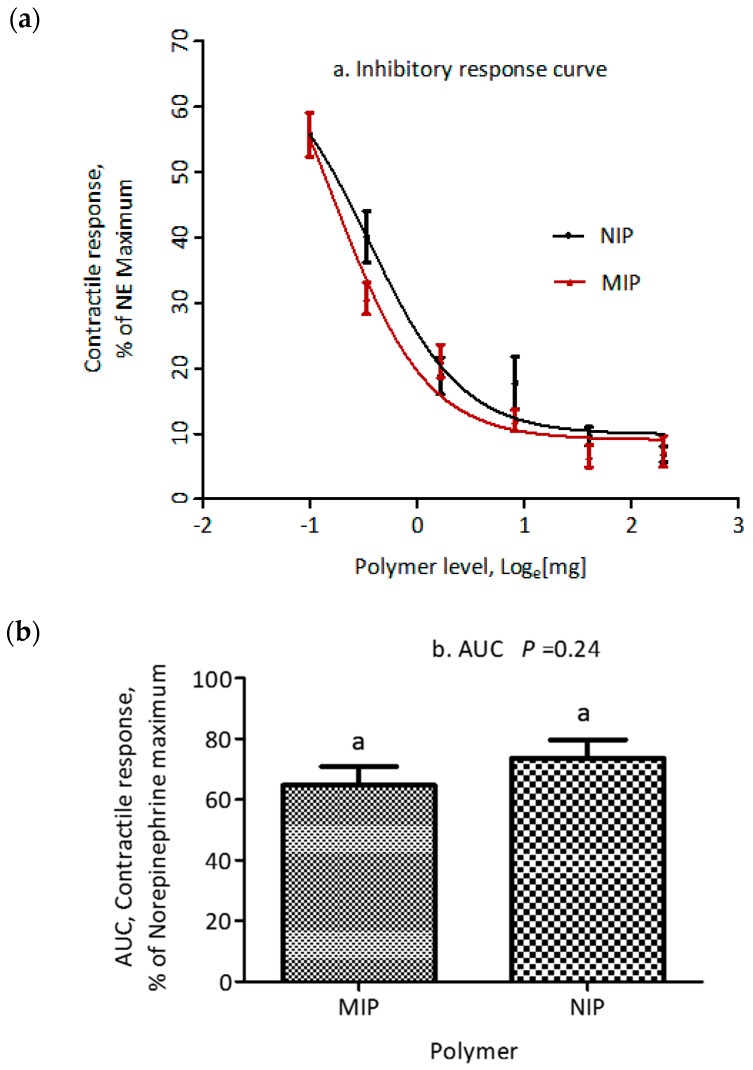
(**a**) Effect of increasing doses of MIP and NIP on the NE-normalized contractile response of saphenous veins, induced by ETA (7.813 × 10^−7^ M) in a 1 h, 39 °C incubation. (**b**) *T*-test analysis of area under the curve for comparison of MIP and NIP. Data are expressed as mean ± SEM (*n* = 12). Similar letters (a) above columns denote lack of significant difference (*p* > 0.05) between columns.

The adsorption properties of polymers to ETA were evaluated using the myographic contractile response. Standard inhibition curves for MIP and NIP are shown in Figure 5a. The smooth muscle contraction of saphenous veins decreased in a dose dependent fashion after ETA was exposed to increasing amounts of each polymer in the range from 0.625 to 10 mg, with IC_50_ of 0.511 and 0.729 mg for MIP and NIP, respectively. However, there was no significant difference (*p* = 0.163) in IC_50_ between MIP and NIP. Additionally, there was no difference in maximum (*p* = 0.96) or minimum (*p* = 0.26) inhibitory response between the polymers tested (Table 3). Furthermore, there was no significant difference (*p* = 0.24) in the calculated area under the inhibitory response curve between MIP and NIP (Figure 5b).

### 2.5. Biological Implications of In Vitro Parameters

To relate the in vitro adsorption parameters to the in vivo biological responses with respect to the adsorption efficacy of the two polymers, the myographic contractile response was estimated for free concentration of ETA at equilibrium from isothermal adsorption studies. Myographic response data were estimated using the equation generated (Equation (1)) from the dose response curve (Figure 1) and corrected by a factor of 0.75, obtained from validation studies for lowered myographic response subsequent to incubation.

NE-normalized % contraction = 0.75 (88.5 + (5.7 − 88.5)/(1 + 10 ^6.664−Free ETA concentration^)) (1)

**Figure 6:**
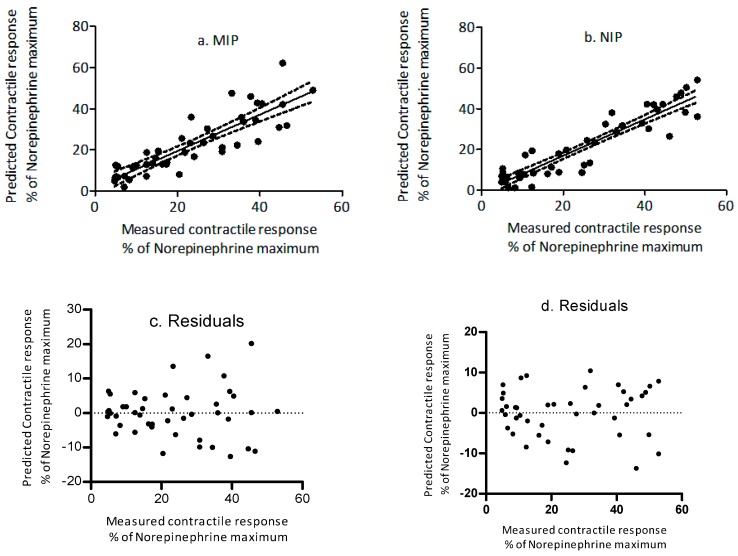
Regression plot between the measured and the predicted percent contractile response (NE-normalized) in the presence of increasing concentrations of (**a**) molecularly imprinted polymer and (**b**) non-molecularly imprinted polymer. Dotted lines represent 95% confidence intervals. The residuals (**c**) MIP and (**d**) NIP are shown on the right side of the plot.

Pre-incubation of ETA in medium containing increasing concentrations of MIP or NIP reduced the contractile response in a dose dependent manner. To determine the biological implications of in vitro adsorption efficiencies, the ex vivo myographic response observed for treatments was related to adsorption efficiencies of polymers, obtained from in vitro studies. The predicted contractile responses determined for free [ETA] in isothermal adsorption studies were regressed against the measured myographic response (Figure 6a,b, Table 4). Results showed that the contractile response for the polymers was predicted with a coefficient of determination of 0.82 and 0.87 for MIP and NIP, respectively. Additionally, for both polymers, the slopes of the prediction lines did not differ (*p* > 0.05) from 1 and the y-intercepts did not differ (*p* > 0.05) from 0.

## 3. Discussion

For grazing animals, one of the most important physiological effects of forage-associated alkaloid consumption is vasoconstriction [5,6,38,39], mediated through effects on smooth muscle, especially via serotonergic [4,42] and adrenergic [43] receptors. Thus, myographic analysis is a relevant tool for assessing the potential value of alkaloid mitigation strategies. We previously developed and characterized a pair of synthetic polymers (MIP and NIP) with demonstrated adsorptive characteristics towards a variety of ergot alkaloids [41]. That work indicated that, in aqueous media, most of the binding occurred through nonspecific interactions, resulting in little difference in binding characteristics towards ETA between MIP and NIP. Evaluation in aqueous media was integral to the present study, given the ultimate interest in the application of adsorbents as feed additives. Maintaining specificity of adsorption in polar solvents is a known challenge with MIP, particularly because of the masking of hydrogen bonding effects important for the specificity of binding [44]. Nonetheless, our previous work indicated comparatively high binding affinities of both of these products for the alkaloid compounds of interest, when compared with other products that have been evaluated in the literature [41]. Additionally, we determined substantial differences in the surface area and porosity of the two products, which could have important effects on binding in different scenarios. The results from the present study corroborated our previous work, in that the isothermal ETA binding characteristics of MIP and NIP were similar to each other. Another goal of the present work was to establish the validity of using simple isothermal adsorption characteristics to predict potential physiological effects after adsorption. This relationship could be confounded by epimerization of ETA, which is influenced by pH, heat and light, resulting in potential differences in the ratio of biologically active (R-) to inactive (S-) forms of ETA [45,46,47]. Although isomeric ratios could be determined analytically (e.g., with LC-MS/MS), extraction conditions (extraction solvents, pH, time of extraction, effects of heat and temperature) would render interpretation challenging. Thus, we utilized a myographic bioassay to determine whether adsorption of ETA onto our synthetic polymers resulted in a quantitatively predictable effect on vasoconstriction.

The initial two validation studies evaluated the influence of incubation time and temperature on the vasoconstrictive response to ETA, and the third validation study confirmed that there was no non-specific binding to the filtration column that would influence the myographic response. Our studies showed a decrease in the myographic response by nearly 25 percent due to incubation, and the reduced contractile response could be due to isomerization to the less active form of the molecule, ergotaminine. Therefore, a correction factor was used to estimate the free vasoactive ETA concentrations following incubation. Additionally, the ETA stock solution was prepared using DMSO to increase the solubility and reduce precipitation in buffer solution. DMSO was present in all the samples in the same proportion.

Our myographic methodology differed somewhat from that of the prior studies which were used to establish the range of ETA concentrations used here. In particular, in the previous studies, the ETA agonist was added in a cumulative fashion to individual vessel sections to establish contractile response curves [7,14]. To avoid potential carryover effects in the present experiment, we used single doses of ETA at different concentrations on each blood vessel section to generate the dose response. Although the shape of the binding curve and binding parameters measured in the present study were similar to those reported for ergot alkaloid exposure in saphenous veins [14], mesenteric arteries and veins [7], and ruminal arteries and veins [6], the lower standard errors in the present work were likely related to this methodological difference.

Compound efficacies are commonly compared using the IC_50_ values, i.e., the inhibitory concentration of the compound that decreases the agonist response by 50 percent [48,49]. The in vitro isothermal adsorption study showed that the amounts of MIP and NIP required to reduce the bioavailability of ETA by 50 percent (*K_d_* values) were 0.51 and 0.57 mg, respectively. When comparing the effect of these polymers in reducing saphenous vein smooth muscle contractions, the efficacies of polymers to reduce the ETA-induced maximal response by 50 percent (IC_50_) were 0.51 and 0.73 mg for MIP and NIP, respectively. Increasing the concentration of either polymer decreased the contractile response, but there was no significant difference between MIP and NIP in reducing the bioavailability of ETA. Although a high affinity of imprinted polymers compared to non-imprinted polymers towards ergot alkaloids has been observed in solid phase extraction (SPE) applications with non-aqueous solvent [50], our results revealed no significant differences for either affinity (*K_d_*) or the IC_50_ values between imprinted and non-imprinted polymers.

Additionally, the measured concentration of ETA remaining after adsorption (i.e., bioavailable concentration) was used to estimate the contractile response using the dose response curve (Equation (3), Section 5.7). With the use of a constant adjustment factor (the 0.75 multiplier in Equation (1), Section 2.5) to account for the systematically lowered contractile response attributed to our incubation model (likely associated with epimerization), vasoconstriction was predicted with reasonable accuracy (*R*^2^ = 0.82 to 0.87) and without bias, for both MIP and NIP. These results indicate that the parameters generated from in vitro studies to determine the efficacy of adsorbents can be extrapolated to their physiological significance in biological systems.

The use of adsorbents to reduce ergot alkaloid bioavailability can be considered more beneficial than strategies attempting to treat the symptoms of ergot alkaloid toxicity. Results of the present study confirm other reports showing a direct relationship between the concentration of ETA and the intensity of the corresponding contractile response of smooth muscles in blood vessels [6,7]. A prolonged and persistent contractile response is induced at low concentrations of ETA if the blood vessel is exposed for an extended period of time [4,14], due to strong interactions with serotonin receptors [5,42]. Additionally, ergopeptides, including ETA and ergovaline, have been shown to interact with serotonergic receptors and myenteric neurons in the gut, affecting gut motility [51,52] and the baseline tone and inhibition of ruminal or reticular contractions [13]. This sustained interaction of ETA with receptors also creates an opportunity for ergot alkaloid accumulation, which may exceed the rate of receptor recycling or turnover, causing greater impact on normal biological processes. Bioaccumulation in lateral saphenous veins after repetitive exposure to ergovaline in vitro has been suggested [53]. The potential for ergot alkaloid build-up in tissues of exposed animals could be one factor behind the persistent contraction and delay in vascular recovery after removal of animals from endophyte infected diets [54,55]. This prolonged stimulation of serotonin receptors may result in receptor desensitization [56] or receptor internalization [57] which may lead to inefficiency with treatments involving antagonistic mechanisms, especially receptor blockers. Therefore, feed additives that reduce the available ergot alkaloids in the gut may provide better protection.

## 4. Conclusions

Ergotamine, along with other ergot alkaloids, can influence vascular smooth contraction of bovine lateral saphenous veins. This study indicated that a pre-myograph incubation with MIP or NIP at an inclusion rate between 0.625 and 10 mg per 7.81 × 10^−7^ M of ETA in 10-mL volume, reduced the contractile response to ETA in lateral saphenous veins in a dose dependent fashion. There were no indications that imprinted polymers were more effective in reducing the contractile response compared to non-imprinted polymers, when evaluated in aqueous environments. The ex vivo contractile response could be predicted from the in vitro adsorption data with more than 80% accuracy, indicating the physiological significance of the isothermal adsorption parameters. These studies indicate that synthetic polymers are potentially effective adsorbents to mitigate ergot toxicity caused by ergot alkaloids with little evidence of significant differences between MIP and NIP. However, further animal studies are required to assess the application of imprinted polymers as selective adsorbents of ergot alkaloids in feed.

## 5. Materials and Methods

The procedures used in this study did not require approval from the University of Kentucky Animal Care and Use Committee because the blood vessels were collected from animals designated for slaughter in a local abattoir or the University of Kentucky Meats Laboratory.

### 5.1. Animals and Tissues

Experiments were conducted using the cranial branch of the lateral saphenous vein, which was dissected from both hind limbs of mixed breed and gender cattle (*n* = 17; BW 572 ± 96 kg; 20–36 months) shortly after the animals were slaughtered (stunned with captive bolt and exsanguinated) at the University of Kentucky or a local abattoir, in accordance with established procedures [38]. Excised tissue sections were placed in a modified Krebs–Henseleit oxygenated buffer (95% O_2_ + 5% CO_2_; pH = 7.4; mM composition = d-glucose, 11.1; MgSO_4_, 1.2; KH_2_PO_4_, 1.2; KCl, 4.7; NaCl, 118.1; CaCl_2_, 3.4; and NaHCO_3_, 24.9; Sigma Chemical Co., St. Louis, MO, USA) that was cooled on ice, and transported to the laboratory. Adipose and connective tissue surrounding the blood vessel was removed and the cleaned vessel was sliced into 2-mm cross-sections under a 12.5× magnifying dissecting microscope (Stemi 2000-C; Carl Zeiss Inc., Oberkochen, Germany), without damaging the endothelium. Before mounting the blood vessels in myograph chambers, the lumen, outer diameter and width of the sections were measured under a light microscope to confirm structural integrity and consistent dimensions across sections (Axiovision, version 20, 2017; Carl Zeiss Inc., Jena, Germany).

### 5.2. Dose Response to ETA

Dose response curves were generated by exposing blood vessels to increasing concentrations of ETA. A stock solution of 20 mM ETA was prepared (11.63 mg/mL DMSO; MW: 581.66, ≥ 97% purity, #45510, Fluka, Sigma Chemical Co.) and aliquoted in 100-μL volumes into silanized HPLC vials and stored at −20 °C until use. A diluting buffer was prepared by adding 500 µL of DMSO to 99.5 mL of modified Krebs–Henseleit buffer. An ergotamine working solution of 1.000 × 10^−3^ M was prepared by adding 100-μL volume of stock solution to 19.90 mL of diluting solvent. Subsequently, ten serial dilutions (1:1) were made using the diluting solution to yield working solutions of 5.000 × 10^−5^, 2.500 × 10^−5^, 1.250 × 10^−5^, 6.250 × 10^−6^, 3.125 × 10^−6^, 1.563 × 10^−6^, 7.813 × 10^−7^, 3.906 × 10^−7^, 1.953 × 10^−7^ and 9.766 × 10^−8^ M, and a control of 0.00 M ETA and a 5-mL aliquot was added to the myograph chamber and incubated for 1 hr at 39 °C. No buffer replacement occurred during this 1-hr incubation period and each blood vessel cross-section was only exposed to a single ETA concentration. At the end of the incubation period, an aliquot of norepinephrine (500 µL, 1 × 10^−4^ M) was added to confirm tissue viability.

### 5.3. Validation Studies

An ergotamine concentration of 7.81 × 10^−7^ M that represented the 75th percentile contractile response from the dose response curve was chosen for all the polymer adsorption studies. The 75th percentile was chosen to ensure that the treatment ETA concentrations, after the addition of polymers, produced a contractile response in the linear range of the sigmoidal dose response curve. Additionally, for accuracy and reliability, the isothermal adsorption conditions, including incubation time (2 min vs. 60 min), temperature during adsorption (39 vs. 21 °C) and non-specific binding (with or without filtration column), that would possibly affect myographic results, were examined and validated.

### 5.4. Polymer Evaluation: Isothermal Adsorption Studies

Methacrylic acid-based polymer adsorbents were synthesized, in accordance with a previously published protocol [40,41]. Briefly, bulk polymerization with self-assembly was used in the synthesis of polymers. During the synthesis of MIP, NIP without template was synthesized using the same protocol. Ergotamine tartrate (5.00 × 10^−3^ mol), methacrylic acid (MAA) (8.00 × 10^−2^ mol) and hydroxyethyl methacrylate (HEMA) (4.00 × 10^−2^ mol) were dissolved in 10 mL toluene in a 250-mL round bottom flask and purged with high-purity dry nitrogen for 30 min at room temperature. Following the incubation period, ethylene glycol dimethacrylate (EGDMA) (4.00 × 10^−1^ mol), 2,2-azobisisobutyronitrile (AIBN) (4.00 × 10^−3^ mol) and toluene (65 mL) were added. Polymerization was initiated by placing the reaction mixture in an oil bath set at 65 °C and propagated for 5-h with stirring. Polymerization was terminated by reducing the temperature to 22 °C. The mixture was then filtered and the pellet was subjected to a template removal procedure using acidic methanol (0.2 N HCL in methanol (100 mL, 5 times) and acetonitrile (100 mL, 5 times). The polymer products were freeze-dried overnight and oven-dried (60 °C for 24 h) before being used in the study.

Isothermal adsorption studies were validated for inclusion rates of polymer that would generate a dose response curve showing affinity and saturation. Two independent studies were conducted, with inclusion rates between 0.001 and 10 mg in log_10_ scale and between 0 and 10 mg in log_2_ scale. Responses from the log_2_ scale yielded a better curve fit, so this scale was used for all polymer evaluations with the myographic studies. Six inclusion levels of polymer were prepared by weighing 0, 0.625, 1.25, 2.5, 5 and 10 mg of MIP or NIP in 30-mL silanized amber color bottles, in duplicate. An ETA working solution was prepared by adding 4.68 μL of stock solution into 120 mL Krebs–Henseleit incubation buffer, yielding 7.813 × 10^−7^ M concentration. A 10 mL volume of ETA working solution (7.813 × 10^−7^ M) was added to each bottle containing different polymer masses and incubated for 15 min with shaking. The solution was then filtered using a solid phase extraction cartridge (SPE) to separate the polymer-ETA complex from free ETA, and a 5 mL aliquot of the filtrate was placed in a water bath for 15 min to reach a temperature of 39 °C. The treatments were added to the myographic chambers and incubated for 1 h at 39 °C. At the end of the incubation period, norepinephrine (500-µL, 1 × 10^−4^ M) was added to confirm tissue viability. Each myograph chamber received only one treatment and treatment concentrations were administered in duplicate within each animal. Additionally, to determine adsorption efficiency, an aliquot of 1 mL from filtrate was collected in a silanized amber HPLC vial and analyzed for ETA concentrations by HPLC-fluorescence.

### 5.5. Myograph Experiments

Prior to mounting blood vessels, 12 myograph chambers (MT610M, Danish Myo Technologies, Atlanta, GA, USA) were calibrated for tension with 2-g calibrated weights. Blood vessel segments were individually mounted on calibrated luminal supports in separate myograph chambers and equilibrated in 5-mL of modified Krebs–Henseleit (37 °C) containing desipramine (3.00 × 10^−5^ M; D3900 Sigma Chemical Co.) and propranolol (1.00 × 10^−6^ M; P0844, Sigma Chemical Co., St. Louis, MO, USA) to inactivate neuronal uptake of catecholamines and to block β-adrenergic receptors, respectively [38]. The buffer solution was replaced at 15 min intervals throughout the equilibration period, for 90 min, to achieve a baseline tension of 1 g. Norepinephrine (500-µL, 1.00 × 10^−4^ M) was added to check the viability of the tissue and for subsequent normalization of the tissue response data. Before the addition of test samples, blood vessels were returned to a baseline tension of 1 g by washing with modified Krebs–Henseleit buffer at 15-min intervals. The maximum tension observed during a 60-min incubation period after the addition of a treatment was recorded and corrected for baseline tension, measured just before the addition of the treatment.

### 5.6. HPLC Analysis of ETA

A series 2695 Alliance HPLC separation module (Waters, Milford, MI, USA), equipped with a binary pump, an auto sampler, a column oven and a fluorescence detector (474 scanning fluorescence detector, λ_ex_ 250 nm, λ_em_ of 420 nm, gain 16 and attenuation 1000) was used to analyze ergot alkloids in the samples. Chromatographic data were integrated using Waters Empower 3 software 7.00.00.99 (Waters, Milford, MI, USA). HPLC separations were performed with a 100 × 4.6 mm i.d., 2.6 µm particle size, Kinetex C18 column (Phenomenex, Torrance, CA, USA) with a gradient elution consisting of two mobile phases, (A) water and (B) acetonitrile, both spiked with ammonium hydroxide (0.04%). Initial gradient conditions were 100% A, held for 1 min, increasing linearly to 100% B over 12 min and held for 3 min. Finally, a linear return to initial conditions over 3 min was held for 2 min for a total run time of 21 min. The sample injection volume was 50 µL. Samples were evaporated to near dryness and the residues dissolved in methanol/water (50/50) and analyzed [34].

### 5.7. Data and Statistical Analyses

The myographic contractile response was recorded at the end of the incubation period, as g of tension, using 16/35 Powerlab and Chart software (Version 8.1.3, 2016; AD Instruments, Colorado Springs, CO, USA). The data were normalized to the maximum contractile response of norepinephrine from the same tissue (Equation (2)), to minimize animal to animal variations and differences in contractile response magnitudes due to different sized veins [38].

Percent contraction = [sample response (Max − Min)/NE response (Max − Min)] × 100
(2)

The minimum and maximum responses of each sample represent the myographic readings (tension in smooth muscle, g), before and after addition of treatment, respectively. For dose response curves, a non-linear standard curve was established by plotting the normalized contractile response against the concentration of ETA, i.e., response vs log [agonist] (Equation (3)).

Y = Min + (Min − Max)/(1 + 10^(LogEC50−X)^) (3)

The EC_50_ (effective concentration) is the concentration of agonist that gives a response half way between the minimum (low contractile response) and maximum (high contractile response), expressed in percent.

To determine the ability of a polymer to reduce the contractile response, the NE-normalized percent contraction of smooth muscle to ETA was fitted against the polymer levels in the media, i.e., response vs log [inhibitor] (Equation (4)).

Y = Min + (Max − Min)/(1 + 10^(X-LogIC50)^) (4)

Maximum (Max) and minimum (Min) are plateaus in the units of the % contraction (NE normalized) and IC_50_ (inhibitory concentration) is the concentration of polymer (mg) that is halfway between maximum and minimum inhibition.

The isothermal adsorption efficiency of polymers to ETA in buffer media was determined using Equation (5) with GraphPad Prism Software, version 5.01 (GraphPad Software 2007, San Diego, CA, USA).

Amount adsorbed (moles·mg^−1^) = (B_max_ × X)/(*K_d_* + X) + NS × X (5)

where, B_max_ is the maximum specific adsorption (moles·specific weight of polymer^−1^), *K_d_* is the equilibrium adsorption constant (mg of polymer), indicating the amount of polymer needed to achieve a half-maximum adsorption at equilibrium and NS is the slope of nonspecific adsorption (moles·mg^−1^).

The percent contractile responses of ETA after the 60-min incubation period were compared between the polymer types. The experimental model used was a completely randomized design and data were analyzed using the mixed procedure of SAS (version 9.2; SAS Inst. Inc., Cary, NC, USA), considering the effect of treatment as a fixed effect, with vein cross-sections serving as the experimental unit. Values are shown as mean ± SEM. Concentration–response curves were fitted by nonlinear regression using response vs log [agonist], and a nonlinear regression of response vs log [inhibitor] was used for polymer evaluation. Statistical analyses for nonlinear fit parameters and for agonist or inhibitor effects on smooth muscle contraction involved simple t-tests using GraphPad Prism Software, version 5.01 (GraphPad Software 2007, CA, USA). Non-normally distributed data analyses were conducted with non-parametric t-tests, followed by the Mann–Whitney test. Differences between means were declared significant for *p*-values lower than 0.05 (in two-tailed tests).

## Figures and Tables

**Table 1:** Dose response parameters for norepinephrine normalized contractile response of saphenous vein smooth muscle to increasing concentrations of ergotamine tartrate (ETA).

Dose Response Parameters ^1^	Mean	95% Confidence Interval
Minimum contractile response, %	5.7	−13.9 to 25.4
Maximum contractile response, %	88.5	80.8 to 96.1
Log EC_50_, −Log [ETA]	6.66	6.32 to 7.01
Goodness of fit		
Degrees of freedom	9	
R square	0.95	

^1^ Experiments were carried out with twelve increasing concentrations of ETA as a single dose exposure to each blood vessel section in individual myograph chambers (*n* = 10). Percent contraction (NE normalized) = 88.5 + (5.7 − 88.5)/(1 + 10^6.664−intital [ETA]^) (Equation (3), Section 5.7). EC_50_, effective molar concentration; NE, norepinephrine.

**Table 2:** Adsorption parameters with polymer to ETA in modified Krebs-Henseleit buffer media at 39 °C (*n* = 10).

	MIP	NIP	*p*-Value
B_max_ (Moles) ^1^	3.27 × 10^−8^ ± 2.391 × 10^−9^	3.15 × 10^−8^ ± 3.877 × 10^−9^	0.78
*K_d_* (mg of polymer) ^2^	0.51 ± 0.108	0.57 ± 0.193	0.80
NS (Moles mg^−1^) ^3^	−2.06 × 10^−10^ ± 2.361 × 10^−10^	−9.63 × 10^−11^ ± 3.776 × 10^−10^	0.81
*R*^2^	0.927	0.835	

^1^ B_max_ is the maximum specific adsorption of ETA in moles at equilibrium. ^2^ *K_d_* is the equilibrium adsorption constant (polymer needed to achieve a half-maximum adsorption). ^3^ NS is the slope of nonspecific adsorption (Moles·mg^−1^).

**Table 3:** Maximum and minimum inhibition and IC_50_ for norepinephrine normalized contractile response of saphenous veins exposed to ETA treated with MIP and NIP.

Contractile Response	Polymers			
	MIP	NIP	SEM	*p*-Value
At maximum inhibition, %	9.1	9.2	1.79	0.96
At minimum inhibition, %	88.0	70.0	10.92	0.26
IC_50_, mg of polymer	0.51	0.73	0.105	0.16

Inhibitory concentration (IC_50_) values were calculated by a fitting concentration-response relationship to a sigmoidal model of the form response vs log [inhibitor] (Equation (4), Section 5.7). The IC_50_ is the concentration of inhibitor (polymer) in milligrams that produces a 50% response between maximum and minimum inhibition.

**Table 4:** Regression equation and fit parameters for the prediction of the contractile response (Y) (NE-normalized) of saphenous vein smooth muscles by ergotamine (x) in the presence of MIP and NIP.

				95% CI	
	Regression Equation	*R*^2^	*p*-Value	Slope	y-Intercept
MIP	Y = 0.98 ± 0.07(x) + 0.15 ± 2.07	0.82	<0.01	0.85 to 1.12	−4.01 to 4.32
NIP	Y = 0.92 ± 0.05(x) − 1.35 ± 1.75	0.87	<0.01	0.81 to 1.03	−4.87 to 2.17
